# Adequate gross resection margin length ensuring pathologically complete resection in gastrectomy for gastric cancer: A systematic review and meta‐analysis

**DOI:** 10.1002/ags3.12761

**Published:** 2023-12-05

**Authors:** Masaru Hayami, Manabu Ohashi, Nozomi Kurihara, Souya Nunobe

**Affiliations:** ^1^ Department of Gastroenterological Surgery, Gastroenterological Center Cancer Institute Hospital, Japanese Foundation for Cancer Research Tokyo Japan; ^2^ Department of Clinical Trial Planning and Strategy Cancer Institute Hospital, Japanese Foundation for Cancer Research Tokyo Japan

**Keywords:** gastrectomy, gastric cancer, negative resection margin, positive resection margin, resection margin length

## Abstract

**Aim:**

A positive resection margin (RM) is associated with poor survival after gastrectomy for gastric cancer (GC). However, the adequate RM length to avoid a positive RM remains controversial. We performed a systematic review to examine the RM length required to avoid a positive RM in gastrectomy for GC.

**Methods:**

This systematic review involved all relevant articles identified in PubMed, the Cochrane Library, Web of Science, and ClinicalTrials.gov until August 2023. The incidence of a positive RM related to the RM length and the possible incidence of a positive RM estimated from the discrepancy between the gross and pathological RM length were evaluated. The Newcastle–Ottawa Scale was used to quantify study quality.

**Results:**

Thirteen studies involving 8983 patients were analyzed. Investigation of the incidence of a positive RM in relation to the RM length showed that a proximal RM length of 6 cm guaranteed a negative RM in gastrectomy. Analyses of the possible incidence of a positive RM revealed that a negative RM would be guaranteed if the proximal RM length was 6 cm in distal gastrectomy, if the esophageal resection length was 2 cm in total gastrectomy for GC without esophageal invasion and 2.5 cm in total or proximal gastrectomy for GC with esophageal invasion or esophagogastric junction cancer, and if the distal RM length was 4 cm in proximal gastrectomy for early GC.

**Conclusions:**

The adequate RM lengths to ensure a pathologically negative RM in each type of gastrectomy for GC were herein suggested.

## Graphical abstract

In gastrectomy for gastric cancer, a pathologically negative resection margin is essential to obtain curative resection because a positive resection margin is associated with poor survival. In this systematic review and meta‐analysis, we examined literature and investigated the adequate resection margin lengths required to avoid a positive resection margin in gastrectomy for gastric cancer.

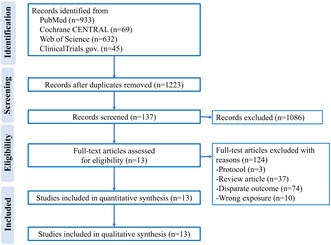

## 1 INTRODUCTION

Gastric cancer (GC) is the fifth most common cancer and the fourth leading cause of cancer‐related death worldwide, with approximately 1 089 100 new diagnoses and 768 800 deaths each year.^1^ Radical gastrectomy, including lymph node dissection to completely eradicate regional tumors, remains the mainstay treatment of curable GC. In gastrectomy for GC, a pathologically negative resection margin (RM) is essential to obtain curative resection because a positive RM is associated with poor survival.^2^, ^3^, ^4^, ^5^, ^6^, ^7^, ^8^, ^9^

Several guidelines have been published on the RM lengths required to avoid positive RMs and, in particular, ensure pathologically negative proximal RMs.^10^, ^11^, ^12^, ^13^, ^14^ In Japan, the Gastric Cancer Treatment Guidelines (GCTGs)^10^ recommend that surgeons should attempt to maintain the proximal RM length at ≥2 cm for cT1 disease, ≥3 cm for cT2 to cT4 (cT2–4) disease of the expansive growth type, and ≥5 cm for cT2–4 disease of the infiltrative growth type (Inf). Furthermore, the GCTGs also recommend submitting the cut edge for intraoperative frozen section (IFS) analysis when the proximal RM length is less than each length recommended in the GCTGs or when a positive RM is suspected. However, the National Comprehensive Cancer Network guidelines^14^ recommend that a gross proximal RM length of ≥4 cm is required to achieve pathologically negative RMs. The guidelines of the European Society for Medical Oncology^13^ also recommend a proximal RM length of ≥5 cm, while advocating 8 cm for diffuse cancers. These different recommendations for the proximal RM length in gastrectomy for GC imply that there is no worldwide standard length. Although a longer RM length is more likely to secure negative RMs, an appropriate length may be ideal. Several systematic reviews have been performed to investigate the predictors of positive RMs and the independent effect of a positive RM or the RM length on survival and recurrence.^15^, ^16^, ^17^ However, the ideal RM length to avoid a positive RM remains controversial.

To determine the adequate RM lengths to avoid a positive RM in gastrectomy for GC, we performed a systematic review of literature regarding the relationship between the gross RM length and pathologically positive RMs.

## 2 METHODS

### 2.1 Search strategy

This study was conducted in accordance with the Preferred Reporting Items for Systematic Reviews and Meta‐Analyses 2020 (PRISMA 2020).^18^ The protocol has been registered on PROSPERO (CRD42023464680).

On 1 August 2023, we searched articles published from January 1970 to August 2023 in the databases of MEDLINE (PubMed), the Cochrane Central Register of Controlled Trials (Cochrane Library), Web of Science, and ClinicalTrials.gov for ongoing or unpublished trials. We retrieved Medical Subject Headings (MeSH) and equivalent text word terms, such as “resection length,” “margin length,” “margin,” “positive margin,” “positive RM,” “positive surgical margin,” “resection line involvement,” “resection margin involvement,” “R1 resection,” “gastric resection,” “gastrectomy,” “GC,” and “stomach neoplasms.”

### 2.2 Study selection

Two investigators (M.H. and M.O.) reviewed the titles and abstracts of all identified studies and judged whether these studies were eligible for review. Both retrospective and prospective cohort studies evaluating the relationship between the gross RM length and pathologically positive RMs in patients undergoing gastrectomy for GC were included. We excluded studies that evaluated risk factors for positive RMs, the prognosis for positive RMs, and the prognosis for the RM length. Abstracts, letters, editorials and expert opinions, reviews without original data, case reports, and studies lacking control groups were excluded. If multiple publications from the same study cohort were presented, the largest and most recently updated datasets were chosen. However, it was deemed acceptable if each publication focused on a different primary endpoint. When a study was considered relevant, we reviewed the full article. The reference lists of all relevant articles were manually reviewed to identify potentially relevant studies. The language for all articles was limited to English.

### 2.3 Data extraction and quality assessment

Two reviewers (M.H. and M.O.) independently reviewed all eligible studies and extracted all relevant data. Discrepancies between the two reviewers were resolved by discussion and consensus. The following data were extracted from each eligible study: type of GC, type of resection, number of patients, RM length, incidence of positive RMs, discrepancy between the gross and pathological RM lengths (ΔRM), and possible incidence of a positive RM estimated from the ΔRM. The Newcastle–Ottawa Scale was used to quantify study quality, and studies achieving six or more stars (out of nine) were considered to be of higher quality.^19^

### 2.4 Statistical methods

The primary outcome of this analysis was to directly evaluate the incidence of pathologically positive RMs associated with each RM length in gastrectomy for GC. The secondary outcome was to evaluate ΔRM and indirectly assess the possible incidence of pathologically positive RMs related to each RM length in gastrectomy. ΔRM was defined as the discrepancy between the gross and pathological tumor boundaries, which shows how long the pathological tumor boundary extends beyond the gross boundary towards the proximal or distal resection line. Thus, we should transect the esophagus or stomach over the ΔRM apart from the gross tumor boundary to obtain a pathologically negative RM. For the analyses of ΔRM, the histograms were constructed with the horizontal axis corresponding to the range of ΔRM length in 5–10 mm increments and the vertical axis corresponding to the number of patients.^20^, ^21^, ^22^, ^23^ Then, based on this histogram, the possible incidence of pathologically positive RMs by respective “gross RM length” in each disease type was calculated or extracted from the studies. The data of studies from our institute for which the actual databases were available were used in the analysis. Heterogeneity across studies was evaluated using the *I* ^2^ test. We considered heterogeneity to be present if the *I* ^2^ statistic was >50%. Outcomes were pooled using a random‐effects model. These results were visualized with a forest plot. All statistical tests were two‐sided, and *p* < 0.05 was considered statistically significant. All statistical analyses were performed using R version 4.3.0 (R Foundation for Statistical Computing, Vienna, Austria).

## 3 RESULTS

### 3.1 Study characteristics

**FIGURE 1:**
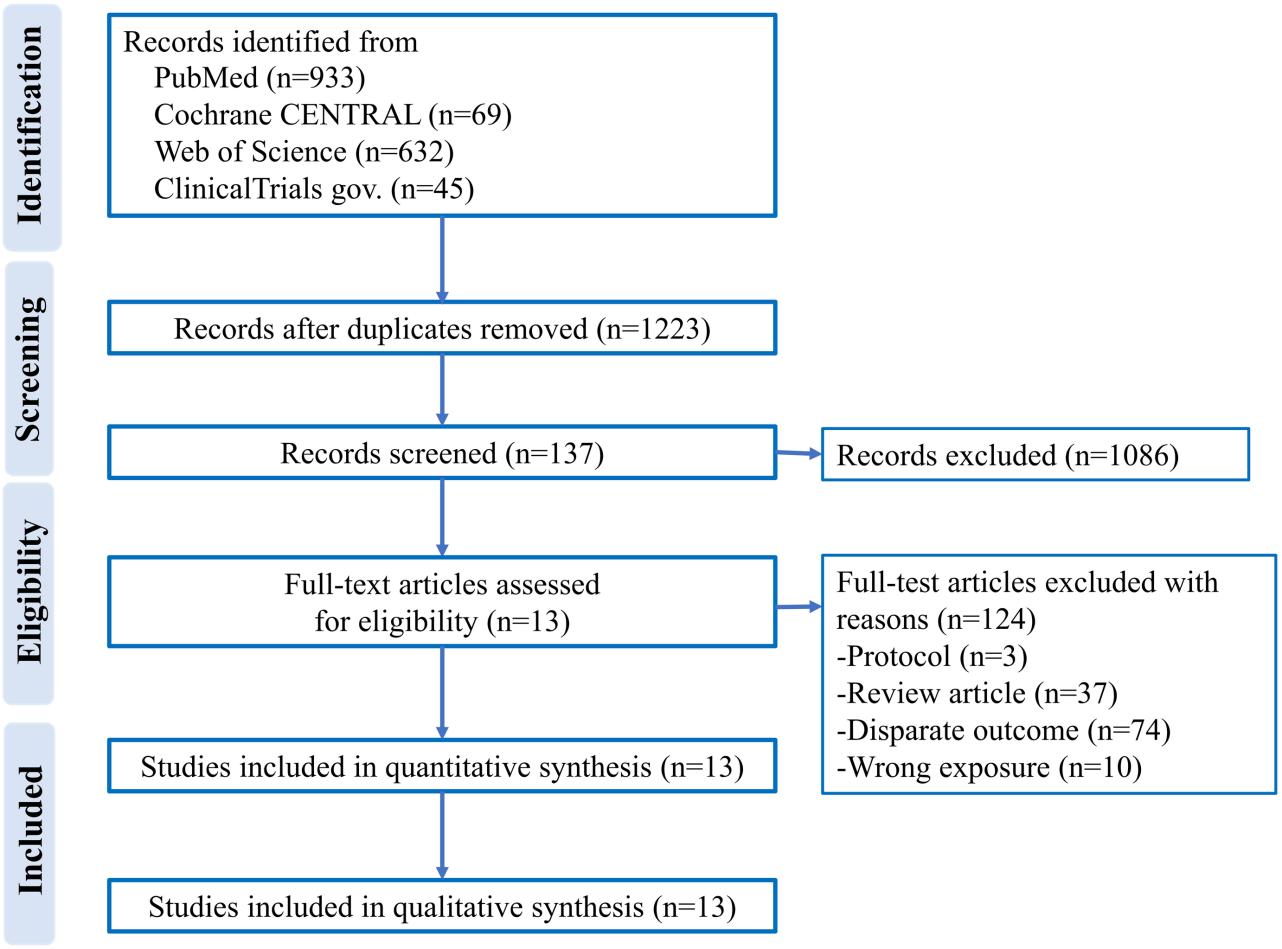
Flowchart of study selection process.

The search strategy initially identified 1223 studies. After exclusion of irrelevant studies, 137 potentially relevant articles were obtained for assessment. We further excluded 124 studies because of the protocol (*n* = 3), review articles (*n* = 37), disparate outcomes (*n* = 74), and irrelevant exposures (*n* = 10). Finally, 13 retrospective studies (seven from Japan, three from South Korea, one from the United States, one from Italy, and one from France) were included.^3^, ^20^, ^21^, ^22^, ^23^, ^24^, ^25^, ^26^, ^27^, ^28^, ^29^, ^30^, ^31^ The PRISMA flowchart of the literature assessment is shown in Figure 1. All 13 studies were methodologically sound with none having <6 stars (Table S1).

### 3.2 Primary outcome

#### 3.2.1 Incidence of pathologically positive RM

Among the 13 retrospective studies, seven studies^3^, ^24^, ^25^, ^26^, ^27^, ^28^, ^31^ involving 4161 patients were available for evaluating the primary outcome. The incidence of a positive RM ranged from 0.2% to 24.4%. All seven studies investigated the proximal RM, and two of them also investigated the distal RM (Table 1). The minimal length to ensure a pathologically negative RM varied among the studies because most studies examined different patients undergoing different types of surgeries. However, a proximal RM length of 6 cm guaranteed a negative RM in gastrectomy for GC.

**TABLE 1:** Incidence of pathologically positive RM in each gross RM length.

Study	Inclusion	Type of resection	Location of RM	Gross RML, cm	Patients, *n*	PRMs, *n* (%)
Papachristou et al.^24^ (1980)	GC (cardia or body)	TG/PG	Proximal	Total	350	73 (20.9)
				0.0	19	19 (100)
				1.0–2.0	72	22 (30.6)
				2.1–4.0	174	26 (14.9)
				4.1–6.0	64	6 (9.4)
				≥6.1	21	0 (0.0)
Bozzetti et al.^25^ (1982)	GC	TG/Gastric resection	Proximal	Total	343	25 (7.3)
				Undetermined	55	11 (20.0)
				0.0	3	3 (100)
				0.1–2.9	98	7 (7.0)
				3.0–5.9	83	4 (4.8)
				≥6.0	104	0 (0.0)
			Distal	Total	343	9 (2.6)
				Undetermined	56	3 (5.4)
				0.0	1	1 (100)
				0.1–2.9	78	3 (3.8)
				3.0–5.9	138	0 (0.0)
				6.0–7.9	34	2 (5.9)
				≥8.0	36	0 (0.0)
Tsujitani et al.^26^ (1995)	GC with EI	TG with EG	Proximal	Total	175	25 (14.3)
				≤0.9	74	16 (21.6)
				1.0–1.9	72	6 (8.3)
				2.0–2.9	21	1 (4.8)
				3.0–3.9	4	1 (25.0)
				≥4.0	4	1 (25.0)
Mariette et al.^3^ (2003)	EGJC (Siewert type I/II/III)	TG/PG with EG (± thoracotomy)	Proximal	Total	94	8 (8.5)
				≤6.0^a^	n.m.	6 (−)
				6.1–7.0^a^	n.m.	2 (−)
				≥7.1^a^	n.m.	0 (0.0)
Ito et al.^27^ (2004)	EGJC (Siewert type II/III)	TG with EG (± thoracotomy)	Proximal	Total	82	20 (24.4)
				Undetermined	11	3 (15.0)
				≤1.9	30	14 (46.7)
				2.0–3.9	9	1 (11.1)
				4.0–5.9	8	2 (25.0)
				≥6.0	24	0 (0.0)
			Distal	Total	82	7 (8.5)
				Undetermined	20	2 (10.0)
				≤1.9	8	3 (37.5)
				2.0–3.9	17	2 (11.8)
				≥4.0	37	0 (0.0)
Kim et al.^28^ (2014)	EGC without EI	sTG/TG	Proximal	Total	2081	5 (0.2)
				≤0.1	5	3 (60.0)
				0.2–1.0	43	0 (0.0)
				1.1–3.0	500	1 (0.2)
				≥3.1	1533	1 (0.07)
Hayami et al.^31^ (2021)	GC	DG	Proximal	Total	1036	8 (0.8)
				≤0.9	6	1 (16.7)
				1.0–1.9	101	4 (4.0)
				2.0–2.9	190	1 (0.5)
				3.0–3.9	158	1 (0.6)
				4.0–4.9	150	1 (0.7)
				≥5.0	431	0 (0.0)

Abbreviations: DG, distal gastrectomy; EGC, early gastric cancer; EG, esophagectomy; EGJC, esophagogastric junction cancer; EI, esophageal invasion; GC, gastric cancer; N.S., not stated; PG, proximal gastrectomy; PRM, positive resection margin; RM, resection margin; RML, resection margin length; sTG, subtotal gastrectomy; TG, total gastrectomy.

^a^ All measurements of the RML were multiplied by two.

Three studies performed subgroup analyses (Table 2). Two of them were classified according to the gross type. Tsujitani et al.^26^ found that a proximal RM length of 2 cm was sufficient to achieve R0 resection in GC with a well‐defined type of esophageal invasion, whereas GC with an ill‐defined type of esophageal invasion could not ensure R0 resection even for a proximal RM length of 4 cm. Hayami et al.^31^ retrospectively analyzed the relationship between adherence to the recommendations of the Japanese GCTGs for the proximal RM length and the positive RM in patients undergoing distal gastrectomy (DG) for GC. They identified no positive RMs in the adherence group for each type of disease.

**TABLE 2:** Subgroup analyses for incidence of pathologically positive RM in each gross proximal RM length.

Study	Inclusion	Type of resection	Subgroup	Gross RML, cm	Patients, *n*	PRMs, *n* (%)
Tsujitani et al.^26^ (1995)	GC with EI	TG with EG	Well‐defined type	Total	63	4 (6.3)
				≤0.9	22	1 (4.5)
				1.0–1.9	32	3 (9.4)
				≥2.0	9	0 (0.0)
			Ill‐defined type	Total	112	21 (18.8)
				0.0	11	9 (81.8)
				0.1–0.9	41	6 (14.6)
				1.0–1.9	40	3 (7.5)
				2.0–2.9	12	1 (8.3)
				3.0–3.9	4	1 (25.0)
				≥4.0	4	1 (25.0)
Ito et al.^27^ (2004)	EGJC (Siewert type II/III)	TG with EG (± thoracotomy)	pT1–2	Total	40	1 (2.5)
				Undetermined	7	1 (14.3)
				≤1.9	9	0 (0.0)
				2.0–3.9	6	0 (0.0)
				4.0–5.9	4	0 (0.0)
				≥6.0	14	0 (0.0)
			pT3–4	Total	42	20 (47.6)
				Undetermined	4	2 (50.0)
				≤1.9	21	14 (66.7)
				2.0–3.9	3	1 (33.3)
				4.0–5.9	4	3 (75.0)
				≥6.0	10	0 (0.0)
Hayami et al.^31^ (2021)	GC	DG	cT1	Total	517	1 (0.2)
				≤1.9	63	1 (1.6)
				≥2.0	454	0 (0.0)
			cT2–4 Exp^a^	Total	158	0 (0.0)
				≤2.9	33	0 (0.0)
				≥3.0	125	0 (0.0)
			cT2–4 Inf^a^	Total	361	7 (1.9)
				≤4.9	225	7 (3.1)
				≥5.0	136	0 (0.0)

Abbreviations: DG, distal gastrectomy; EG, esophagectomy; EGJC, esophagogastric junction cancer; EI, esophageal invasion; Exp, expansive growth type; GC, gastric cancer; Inf, infiltrative growth type; PRM, positive resection margin; RM, resection margin; RML, resection margin length; TG, total gastrectomy.

^a^ According to the Japanese Classification of Gastric Carcinoma.

Ito et al.^27^ investigated the incidence of positive RMs according to the RM length and pT status in patients with esophageal invasion or esophagogastric junction (EGJ) cancer (EGJC) (Siewert type II or III). In their study, no cancer infiltration was observed in RMs for which the gross proximal and distal RM lengths were ≥6 cm in pT3–4 disease and 4 cm, respectively.

#### 3.2.2 Meta‐analyses

**FIGURE 2:**
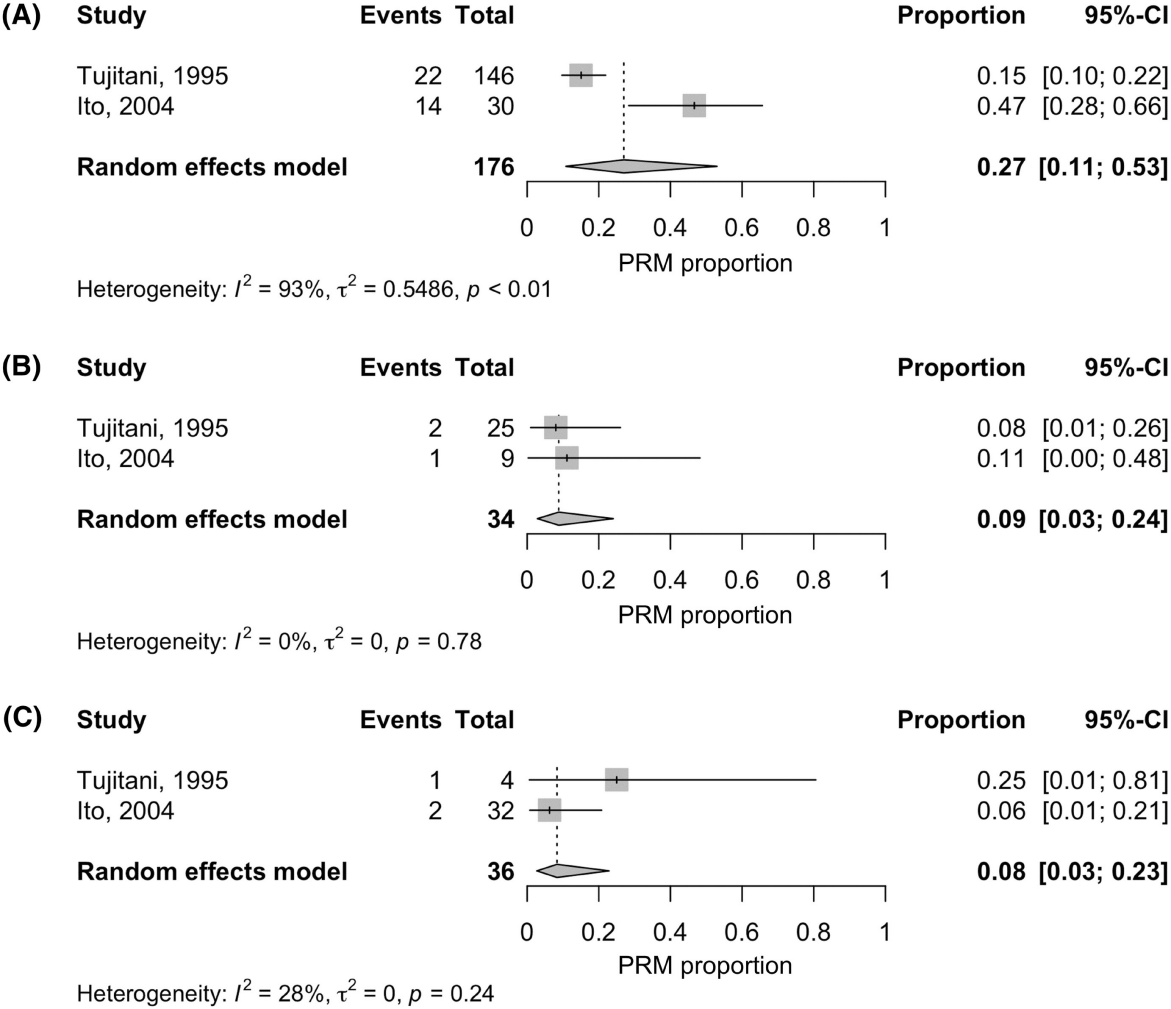
Forest plot of studies investigating the incidence of positive RM related to gross proximal RM length. (A) Proximal RM length of <2 cm. (B) Proximal RM length of 2 to <4 cm. (C) Proximal RM length of ≥4 cm. CI, confidence interval; PRM, positive resection margin; RM, resection margin.

Meta‐analyses were able to be performed for only two similar studies that investigated the incidence of positive RMs in total gastrectomy (TG) with esophagectomy for GC with esophageal invasion or EGJC.^26^, ^27^ The results suggested that the estimated pooled incidences of a positive RM were 27% (95% confidence interval, 11%–53%), 9% (3%–24%), and 8% (3%–23%) for a proximal RM length of <2, 2 to <4, and ≥4 cm, respectively (Figure 2).

### 3.3 Secondary outcome

#### 3.3.1 Possible incidence of pathologically positive RM

Among the 13 retrospective studies, six studies^20^, ^21^, ^22^, ^23^, ^29^, ^30^ involving 4822 patients were available for analyzing the secondary outcome. The characteristics of the six studies are shown in Table 3. These studies evaluated the ΔRM (discrepancy between the gross and pathological RM lengths in gastrectomy), including four studies^20^, ^21^, ^22^, ^23^ from our institute that evaluated the possible incidence of a pathologically positive RM associated with each ΔRM. We also adapted the same concept in the remaining two studies,^29^, ^30^ both from South Korea, of which Berlth et al.^30^ evaluated the ΔRM in the proximal RM for pT2–4 disease. These studies revealed that a negative RM would be guaranteed by a proximal RM length of 6 cm in DG, esophageal resection length of 2 cm in TG for GC without esophageal invasion, and 2.5 cm in TG or proximal gastrectomy (PG) for GC with esophageal invasion or EGJC. Furthermore, a distal RM length of 4 cm guaranteed a negative RM in PG for early GC.

**TABLE 3:** Possible incidence of pathologically positive RM in each gross RM length.

Study	Inclusion	Type of resection	Location of RM	Patients, *n*	Gross RML, cm								
					≥0.0	≥1.0	≥1.5	≥2.0	≥2.5	≥3.0	≥4.0	≥5.0	≥6.0
Choi et al.^29^ (2017)	EGC	DG/PPG/PG/TG	Proximal or distal	861	78.4			10.1					
Berlth et al.^30^ (2020)	pT2–4 GC	DG	Proximal	1033		10.3		5.7		2.0	1.5	1.2	0.0
Hayami et al.^20^ (2020)	GC	DG	Proximal	1273	76.0	14.8		4.3		1.8	0.7	0.2	0.0
Koterazawa et al.^21^ (2022)	Upper EGC	PG	Distal	361		11.6		1.7		0.3	0.0		
Koterazawa et al.^22^ (2022)	GC without EI	TG	Proximal (ERL)	1005	9.8	0.8		0.0					
Koterazawa et al.^23^ (2023)	GC with EI/EGJC	TG/PG	Proximal	289		13.1	5.5	0.3	0.0				

Abbreviations: DG, distal gastrectomy; EGC, early gastric cancer; EGJC, esophagogastric junction cancer; EI, esophageal invasion; ERL, esophageal resection length; GC, gastric cancer; PG, proximal gastrectomy; RM, resection margin; RML, resection margin length; TG, total gastrectomy.

Additionally, four studies^20^, ^21^, ^22^, ^23^ from our institute performed subgroup analyses (Table 4). Hayami et al.^20^ determined the minimum gross proximal RM lengths with zero risk of positive RMs in DG according to the tumor depth, pathological differentiation, and growth type. In the same way, Koterazawa et al.^21^ determined the minimum gross distal RM lengths with zero risk of positive RMs in patients undergoing PG for early GC according to the tumor size and pathological differentiation. They also determined the esophageal resection length to ensure a negative RM for GC with esophageal invasion or EGJC^23^ according to the tumor size and growth type, and for GC without esophageal invasion^22^ according to the tumor size and location (length between the proximal tumor boundary and the EGJ).

**TABLE 4:** Subgroup analyses for possible incidence of pathologically positive RM in each gross RM length.

Study	Inclusion	Type of resection	Location of RM	Subgroup		Patients, *n*	Gross RML, cm								
							≥0.0	≥1.0	≥1.5	≥2.0	≥2.5	≥3.0	≥4.0	≥5.0	≥6.0
Hayami et al.^20^ (2020)	GC	DG	Proximal	cT1 Dif^a^		259	79.2	10.8		0.0					
				cT2–4 Exp^a^		194	73.2	6.7		1.5		0.0			
				cT1 Und^a^		330	78.2	12.4		3.0		0.9	0.0		
				cT2–4 Inf^a^, cTumor size	<8 cm	384	72.9	18.2		6.0		2.3	0.8	0.0	
					≥8 cm	106	78.3	34.0		17.9		10.4	5.7	2.8	0.0
Koterazawa et al.^21^ (2022)	Upper EGC	PG	Distal	cT1 Dif^a^, cTumor size	<1.5 cm	28		0.0							
					>1.5 to 5 cm	140		11.4		0.0					
					>5 cm	22		31.2		9.1		0.0			
				cT1 Und^a^		171		11.1		2.3		0.6	0.0		
Koterazawa et al.^22^ (2022)	GC without EI	TG	Proximal (ERL)	cT1, PB‐EGJ length	>1 cm	207	0.0								
					0 to 1 cm	70	20.0	0.0							
				cT2–4 Exp^a^, PB‐EGJ length	>1 cm	147	0.0								
					0 to 1 cm	49	32.7	0.0							
				cT2–4 Inf^a^, PB‐EGJ length	>3 cm	148	0.0								
					>1 to 3 cm	239	3.3	0.0							
					0 to 1 cm	70	41.3	5.5		0.0					
Koterazawa et al.^23^ (2023)	GC with EI/EGJC	TG/PG	Proximal	cTumor size	≤4 cm	102		2.0	0.0						
					>4 cm	187		19.3	8.6	0.5	0.0				
				Sup^a^		55		1.8	0.0						
				Exp^a^		87		4.6	2.3	0.0					
				Inf^a^		147		22.4	9.5	0.7	0.0				

Abbreviations: cTumor size, gross tumor size; DG, distal gastrectomy; Dif, differentiated type; EGC, early gastric cancer; EGJC, esophagogastric junction cancer; EI, esophageal invasion; ERL, esophageal resection length; Exp, expansive growth type; GC, gastric cancer; Inf, infiltrative growth type; PB‐EGJ length, length between proximal tumor boundary and esophagogastric junction; PG, proximal gastrectomy; RM, resection margin; RML, resection margin length; Sup, superficial growth type; TG, total gastrectomy; Und, undifferentiated type.

^a^ According to the Japanese Classification of Gastric Carcinoma.

#### 3.3.2 Meta‐analyses

**FIGURE 3:**
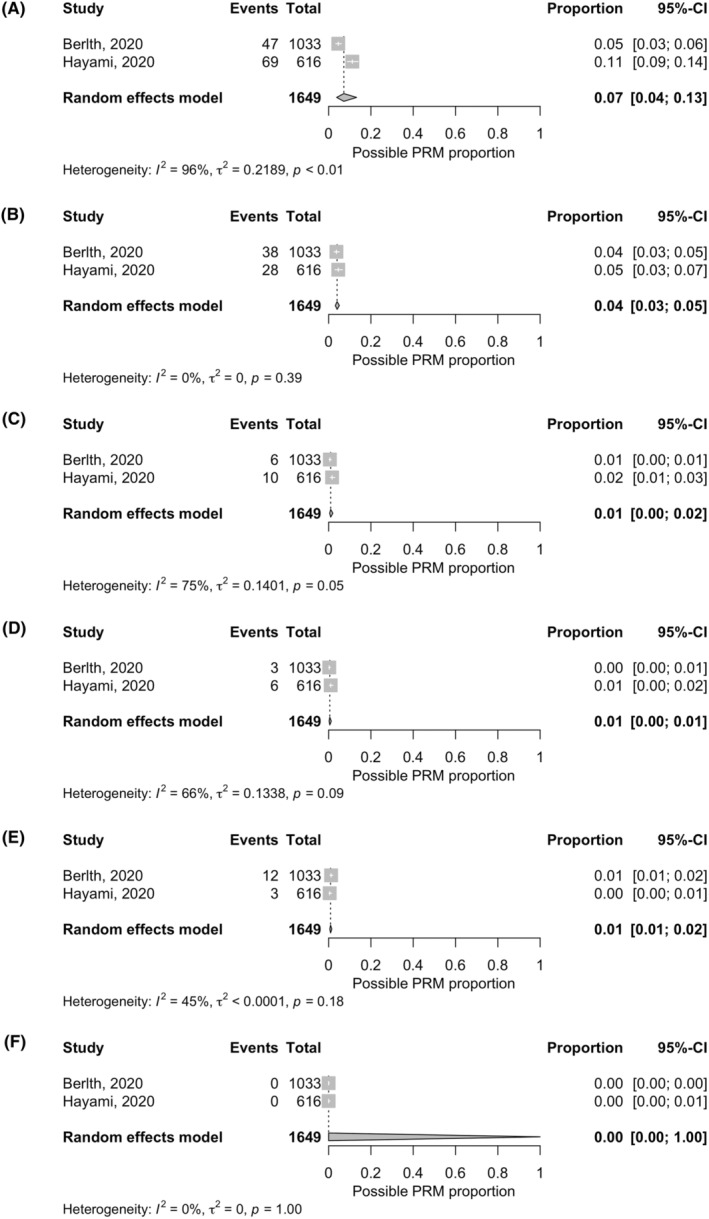
Forest plot of studies investigating the possible incidence of positive RM related to gross proximal RM length. (A) Proximal RM length of 1 to <2 cm. (B) Proximal RM length of 2 to <3 cm. (C) Proximal RM length of 3 to <4 cm. (D) Proximal RM length of 4 to <5 cm. (E) Proximal RM length of 5 to <6 cm. (F) Proximal RM length of ≥6 cm. CI, confidence interval; PRM, positive resection margin; RM, resection margin.

Meta‐analyses were performed in two similar studies^20^, ^30^ that investigated the proximal ΔRM in DG for pT2–4 disease. In the study by Hayami et al.,^20^ the patients with pT2–4 disease were extracted from the dataset and evaluated to match their backgrounds with those of the patients in the study by Berlth et al.^30^ The results suggested that the estimated pooled possible incidence of positive RM was 7% (95% confidence interval, 4%–13%), 4% (3%–5%), 1% (0%–2%), 1% (0%–1%), 1% (1%–2%), and 0% (0%–100%) with a gross proximal RM length of 1 to <2, 2 to <3, 3 to <4, 4 to <5, 5 to <6, and ≥6 cm, respectively (Figure 3).

### 3.4 Publication bias

Publication bias was not assessed because of the small number of included studies available for assessment of the primary and secondary outcomes (fewer than 10).

## 4 DISCUSSION

The present analyses revealed the incidence or possible incidence of positive RMs related to RM lengths in gastrectomy for GC. Although the number of included studies was limited, to the best of our knowledge, this is the first review and meta‐analysis to focus on the RM length to ensure negative RMs in gastrectomy for GC. The results of our study will serve as a useful reference both preoperatively and intraoperatively to determine the adequate RM length with which to avoid positive RMs in patients undergoing gastrectomy for GC. Furthermore, the estimated incidence of a positive RM for each RM length and procedure was directly or indirectly presented. Surgeons can refer to these results to determine how frequently a positive RM occurs with each RM length. If the incidence of a positive RM is very low, gastrectomy with a short RM length that does not ensure a pathologically negative RM may be acceptable.

Numerous authors have identified risk factors for positive RMs; these risk factors are mainly associated with more aggressive tumor biology, including a larger tumor size, higher tumor location, advanced TNM status, undifferentiated or diffuse histological type, higher Borrmann type, and lymphovascular invasion.^2^, ^4^, ^6^, ^7^, ^8^, ^32^, ^33^, ^34^, ^35^, ^36^, ^37^, ^38^, ^39^ Thus, IFS analysis should be routinely performed in patients who have such risk factors to reduce the incidence of positive RMs and determine the ideal gastric or esophageal transection line. Although shorter RM lengths seem to increase the risk of positive RMs, specific RM lengths for avoiding positive RMs have not yet been sufficiently discussed. Among the papers discussing risk factors for positive RMs, only Bissolati et al.^37^ identified the RM length as an independent risk factor for a positive RM. However, how long surgeons should actually maintain RM lengths in gastrectomy has not been thoroughly analyzed.

A proximal RM length of 6 cm to ensure a negative pathological RM in gastrectomy for GC obtained in the present systematic review may be a reliable result because four of the 13 studies and the meta‐analyses of the secondary outcome produced this same finding.^20^, ^24^, ^25^, ^30^ However, 6 cm is relatively long when GC is located in the middle to upper third of the stomach. If surgeons strictly maintain the suggested RM length, TG should be selected in many patients even though they have early GC. The subgroup analyses of the primary and secondary outcomes were useful to address this problem. We found that the recommendations of the Japanese GCTGs for DG were appropriate.^20^, ^31^ Additionally, the subgroup analysis showed that cT1 undifferentiated type and cT2–4 Inf of >8 cm requires an RM length of 4 and 6 cm, respectively.^20^

For TG, determining an adequate RM length is slightly complicated, especially in GC without esophageal invasion. The transected organ is the esophagus in such cases, and it may be inadvisable to combine DG and TG when attempting to determine an adequate RM length. Surgeons never transect the esophagus apart from the proximal boundary of the tumor during TG as they do in DG. For instance, although cT2–4 Inf of >8 cm is located in the upper stomach, the proximal boundary of which is immediately below the EGJ, surgeons never transect the esophagus 6 cm away from the EGJ. Thus, the proximal RM length required to ensure a negative pathological RM in TG for GC without esophageal invasion should be analyzed only in patients undergoing TG or PG (procedures in which the esophagus is transected). Only one of 13 studies satisfied this requirement.^22^ In that study, unexpected pathological esophageal extension was newly considered and suggested esophageal resection lengths in several subgroups were determined using ΔRM in the esophagus. We believe that these results are the most reliable in determining the optimal proximal RM length of the esophagus for TG or PG.

The primary outcome for GC with esophageal invasion or EGJC revealed that the proximal RM length must be ≥6 cm to ensure a negative RM, and the subgroup analyses showed that tumor extension varied according to the gross type and pT status. However, these studies might have included patients who had EGJC with long esophageal invasion, and they might have undergone subtotal esophagectomy. Furthermore, the number of patients in each subgroup was very small. Thus, the results are less reliable. Koterazawa et al.^23^ limited their study to patients whose esophageal invasion was <4 cm, for which transhiatal resection is acceptable. For such patients, they suggested a proximal RM length of 2.5 cm. Furthermore, they performed a subgroup analysis and suggested an adequate proximal RM length for each disease. In surgery for GC with esophageal invasion or EGJC, the esophageal resection length is critical because a long esophageal resection length is sometimes required, which usually influences the surgical difficulty. Thus, the suggestions for specific subgroups may be very useful in daily practice.

Few studies provided information regarding the distal RM length in this review. Essentially, because the transected organ in DG or TG is always the duodenum, the distal RM length strongly depends on the tumor location. Unless the GC is close to the pylorus, determining the ideal distal RM length almost does not merit discussion. Thus, the studies that analyzed the distal RM length in DG or TG did not provide valuable information. Only the study by Koterazawa et al.,^21^ in which the distal RM length was analyzed in PG for early GC, was useful in determining adequate RM lengths.

This study may provide surgeons helpful information for determining an adequate RM length for each disease or procedure. Although the primary endpoint of this study was determining RM lengths that ensured pathologically negative RMs, this study also revealed the estimated incidence of positive RMs according to each RM length. Surgeons can refer to these results to determine the optimal gastric or esophageal transection site, although the incidence of a positive RM is not zero. For instance, according to the meta‐analysis of the secondary outcome, the estimated pooled possible incidence of a positive RM is only 1% when the stomach is transected ≥3 cm from the proximal boundary of pT2–4 disease. Surgeons can transect the stomach 3 cm from the proximal boundary and submit the RM for IFS analysis instead of maintaining an RM length of 6 cm, which may avoid TG. Surgeons do not always need to maintain the suggested RM length. Maintaining a shorter RM length and submitting the specimen for IFS analysis considering the estimated incidence of a positive RM for each RM length is acceptable.

Another controversial issue regarding the RM length is its true impact on patient survival. Since the 1980s, many studies have revealed that short RM lengths are associated with higher recurrence rates and poorer survival.^40^, ^41^, ^42^, ^43^ However, we must interpret these findings with caution for two main reasons. First, the RM length tends to be pathologically short in patients with advanced disease, in whom poor survival is expected. This means that the malignant potential of the disease, which is sometimes characterized by unexpected local extension and a short pathological RM length, could affect patient survival and thereby act as a confounding factor when assessing the correlation between the short RM length and adverse outcomes. Second, studies that included patients with inadequately short RM lengths or positive RMs presented poor survival. The poorer survival outcomes of patients with an inadequate RM length in such studies might be attributed to patients with a positive RM, which represented R1 resection. By contrast, several studies revealed that the RM length did not affect survival outcomes.^28^, ^30^, ^44^, ^45^, ^46^, ^47^ These studies showed that gross or pathological RM lengths that were negative for cancer had no effect on survival. Thus, various controversial aspects remain regarding the true impact of the RM length on survival. Surgeons must ensure at least pathologically negative RMs to avoid survival disadvantages until the impact of the RM length on survival outcomes is truly revealed.

This analysis had several limitations. First, all studies included in this analysis were retrospective and uncontrolled, which might have introduced selection bias. In particular, with regard to the secondary outcomes shown in Tables 3 and 4, all studies from Japan finally extracted were from our institute only. Although we followed certain selection criteria of systematic review, this could be a heavy bias affecting the outcomes, which lowered the level of evidence. Second, the small number of studies included in the meta‐analysis, coupled with the limited number of patients in individual studies, did not allow for a highly accurate meta‐analysis. In addition, although we applied random‐effects models, the heterogeneity could not be ignored. Third, this review investigated the incidence of positive RMs related to the RM lengths and did not include studies that evaluated the RM length based on the survival outcome. This is because, as mentioned above, the relationship between the RM length and survival remains controversial. However, patient survival is the most crucial aspect, and further investigation is required to determine the optimal RM length that takes into account both the incidence of positive RMs and the survival outcomes.

## 5 CONCLUSIONS

Based on our systematic review and meta‐analyses, we suggest the following RM lengths in each type of gastrectomy for GC or EGJC: proximal RM length of 6 cm in DG, esophageal resection length of 2 cm in TG for GC without esophageal invasion and 2.5 cm in TG or PG for GC with esophageal invasion or EGJC, and distal RM length of 4 cm in PG for early GC. A shorter RM length for each group in daily practice may be acceptable according to the subgroup analyses and the data on the incidences of the estimated pathologically positive RMs. Despite the inherent limitations, the present systematic review and meta‐analysis may help surgeons to scientifically determine where they should transect the stomach or esophagus for various types of GC and procedures.

## AUTHOR CONTRIBUTIONS

Conception and design: MH and MO. Provision of study materials or patients: MH and MO. Collection and assembly of data: MH and MO. Data analysis and interpretation: MH, MO, and NK. Manuscript writing: All authors. Final approval of the manuscript: All authors.

## FUNDING INFORMATION

The authors declare that no external funding was received for this study.

## ethical statement

Ethical Approval: No ethical approval or informed consent statement was required for this review article.

## CONFLICT OF INTEREST STATEMENT

The authors declare no conflicts of interest.

## Supporting information


Table S1.
