# Supplementary material for: Adequate gross resection margin length ensuring pathologically complete resection in gastrectomy for gastric cancer: A systematic review and meta‐analysis
Source: Ann Gastroenterol Surg. 2023 Dec 5;8(2):202–13. doi: 10.1002/ags3.12761 (PMC10914694; doi:10.1002/ags3.12761)
Supplement: Supplementary file 1 — Table S1. [file AGS3-8-202-s001.docx]

**Supplementary Table 1. NOS quality assessment of included studies**

| **Study** | **Representativeness of exposed cohort** | **Selection of nonexposed cohort** | **Ascertainment of exposure** | **Outcome not present at start of study** | **Comparability** | **Assessment of outcomes** | **Length of follow-up** | **Adequacy of follow-up** | **Total** |
| --- | --- | --- | --- | --- | --- | --- | --- | --- | --- |
| Papachristou et al.^24^ (1980) | 1 | 1 | 1 | 1 | 1 | 1 | 1 | 1 | 8 |
| Bozzetti et al.^25^ (1982) | 1 | 1 | 1 | 1 | 1 | 1 | 1 | 1 | 8 |
| Tsujitani et al.^26^ (1995) | 1 | 1 | 1 | 1 | 1 | 1 | 1 | 1 | 8 |
| Mariette et al.^3^ (2003) | 1 | 1 | 1 | 1 | 1 | 1 | 1 | 1 | 8 |
| Ito et al.^27^ (2004) | 1 | 1 | 1 | 2 | 1 | 1 | 1 | 1 | 9 |
| Kim et al.^28^ (2014) | 1 | 1 | 1 | 1 | 0 | 1 | 1 | 1 | 7 |
| Choi et al.^29^ (2017) | 1 | 1 | 0 | 1 | 1 | 0 | 1 | 1 | 6 |
| Berlth et al.^30^ (2020) | 1 | 1 | 1 | 1 | 1 | 1 | 1 | 1 | 8 |
| Hayami et al.^20^ (2020) | 1 | 1 | 1 | 1 | 0 | 1 | 1 | 0 | 6 |
| Hayami et al.^31^ (2021) | 1 | 1 | 1 | 1 | 2 | 1 | 1 | 1 | 9 |
| Koterazawa et al. ^21^ (2022) | 1 | 1 | 1 | 1 | 0 | 1 | 1 | 0 | 6 |
| Koterazawa et al. ^22^ (2022) | 1 | 1 | 1 | 1 | 0 | 1 | 1 | 0 | 6 |
| Koterazawa et al.^23^ (2023) | 1 | 1 | 1 | 1 | 1 | 1 | 1 | 0 | 7 |

NOS, Newcastle–Ottawa Scale
